# Burden of malaria-HIV coinfection and associated adverse outcomes in Ethiopia: a systematic review and meta-analysis

**DOI:** 10.1186/s12879-026-13609-8

**Published:** 2026-05-19

**Authors:** Temesgen Mitiku Yeshanew, Bokretsion Gidey, Betelhem Abebe, Gemechis Waktole, Birhan Getie, Temesgen Getu, Nega Birhane

**Affiliations:** 1 Department of Biotechnology, Dambi Dollo University, P.O. Box 260, Dambi Dollo, Ethiopia; 2 Department of Medical Biotechnology, Institute of Biotechnology, University of Gondar, P.O. Box 196, Gondar, Ethiopia; 3 Ethiopia Public Health Institute, P.O. Box 1242, Addis Ababa, Ethiopia

**Keywords:** Malaria, HIV, Coinfection, Severe anemia, Parasite load, CD4 count

## Abstract

**Background:**

The co-occurrence of malaria and HIV represents a significant health crisis in sub-Saharan Africa, particularly in Ethiopia, where both diseases are prevalent. This synergistic burden aggravates health inequalities, strains healthcare infrastructure, and challenges socioeconomic stability. Investigating the relationship between these co-infections is crucial for enhancing treatment, prevention, diagnosis, and control strategies for malaria in individuals living with HIV. Therefore, this systematic review and meta-analysis aimed to determine the overall burden of malaria-HIV co-infection, as well as the rates of severe anemia, hyperparasitemia, and CD4 + T-cell counts.

**Methods:**

A comprehensive literature search was conducted using several electronic databases, including PubMed, Science Direct, Scopus, and Cochrane, as well as search engines like Google Scholar. A random-effects model was used to calculate pooled burden estimates. Subgroup analysis and funnel plots were utilized to evaluate heterogeneity and publication bias, respectively.

**Result:**

Of the 429 studies initially reviewed, ten articles met the criteria for inclusion in the final analysis. The pool prevalence estimate of malaria-HIV co-infection was 9% (95% CI 3–15). When grouped by the year of conduct, studies from 2013 to 2025 exhibited the highest malaria burden at 10% (95% CI -4-24), while those conducted from 2002 to 2012 showed the lowest burden at 9% (95% CI 1–16). The overall combined prevalence rates for specific clinical conditions were 31% (95% CI 12–50) for severe anemia, 41% (95% CI -8-90) for hyperparasitemia, and 35% (95% CI 14–56) for low CD4 counts (≤ 200 cells/µL).

**Conclusion:**

This meta-analysis found that the overall burden of malaria-HIV co-infection in Ethiopia is 9%.

## Introduction

Approximately 263 million cases of malaria were reported in 2023 in 83 countries where the disease is endemic, an increase of 11 million cases over 2022 [1]. *P. falciparum* was the most common species in the WHO African Region, where approximately 94% of all cases occurred. Furthermore, 597,000 malaria-related deaths have been reported [2, 3]. A major obstacle to eradicating malaria is the diverse features of *Plasmodium* species, which are regional differences in disease severity, transmission efficiency, and antimalaria drug efficacy [4–6]. While more than 200 species of *Plasmodium* exist as agents of malaria, just five of these species are implicated in causing malaria in human hosts [7]. The pathophysiological progression of severe *P. falciparum* can result in several significant complications, including sequestered parasites that cause cerebral malaria, acute kidney injury due to hemoglobinuria, metabolic dysregulation leading to hypoglycemia [8] and extensive hemolysis resulting in severe anemia. The other major obstacle to eradicating malaria is also vector adaptability, specifically the ability of Anopheles mosquitoes to develop physiological and behavioral resistance to insecticides [9].

The concurrency of malaria and HIV infection presents a substantial hurdle for public health efforts throughout the tropics [10, 11]. Malaria exacerbates HIV in co-infected individuals by triggering an inflammatory response. This inflammation boosts the viral load and creates an optimal setting for HIV replication [12, 13], which subsequently causes a reduction in CD4 cell counts [14, 15]. When CD4 + T cells drop below the critical threshold of 200 cells/µL, the host becomes significantly more susceptible to opportunistic coinfections, which, in turn, boosts overall AIDS-related illness and death rates [16, 17]. HIV/AIDS patients with associated malaria infections are high risk for increased malaria parasite load, low birth weight, stillbirth, anemia, immune dysregulation, severe malaria development [18], treatment fail [19], and the emergence of drug-resistant malaria strains [20].

The coexistence of malaria and HIV presents a risk factor that can lead to severe anemia, significantly impairing a patient’s quality of life and clinical prognosis [15, 21]. Individuals living with HIV/AIDS who are also infected with *Plasmodium* face a greater risk of being anemic or severely anemic than do HIV/AIDS patients who are not infected with malaria [22, 23]. Previous studies conducted in different areas revealed that malaria and HIV coinfection increases the risk of developing anemia, with rates of 48.8% [24], 91.3% [25], and 47.8% [26]. Another meta-analysis of 23 studies confirmed that the incidence of anemia increased by 49% in co-infected pregnant women compared with those with HIV alone [27]. Data suggest that in individuals with malaria and HIV coinfection, the parasitic load is greater than that in individuals with a single infection [24, 28]. Malaria-HIV coinfection is also increasing the parasitic load. Different researchers have confirmed that coinfection causes an increase in parasite loads; as reported, the +++ (1,000–10,000/µL) count was 47.2% [29], and a meta-analysis revealed that the pooled frequency of severe malaria was 43% [30].

Despite the high rates of malaria and HIV across sub-Saharan Africa, there is insufficient data regarding the synergistic and bidirectional interactions between these two infections. Reports indicate a high prevalence of malaria coinfection: 29.4% in Cameroon [31] and Congo (20.33%) [32] 32% in Nigeria [33], and 17.4% in Northwest, Ethiopia [25]. Those studies consistently identify coinfection as a predictor of anemia. While the predictive role of drug resistance in the region has not yet been confirmed, it is anticipated. Given the substantial geographic overlap of high malaria and HIV prevalence, growing evidence indicates that these infections synergistically exacerbate each other. This interaction increases incidence rates and complicates treatment efforts. The rising rates of malaria-HIV coinfection are placing a growing burden on public health systems. Unfortunately, there is limited information on how these infections interact at the molecular and biological levels. This lack of understanding directly hampers efforts to diagnose, treat, prevent, and control malaria in HIV-positive patients. The main objective of this systematic review and meta-analysis was to determine the pooled burden of malaria-HIV co-infection in Ethiopia. In addition, estimating the variation in the prevalence of severe anemia, parasitaemia, and low CD4 cell count in Ethiopia is another objective of this meta-analysis.

## Methods

### Study area

This meta-analysis utilized data from Ethiopia, a nation known for its significant topographic diversity, with elevations ranging from 110 m below sea level to 4,550 m above sea level. The predominant climate is a tropical wet and dry climate characterized by lowland, midland, and highland agro-ecological regions [34]. The mean annual temperatures vary significantly: the highlands experience temperatures between 10 °C and 16 °C, the midlands range from 16 °C to 29 °C, and the lowlands fall within the 23° to 33 °C range. The annual rainfall in highlands ranges from 500 mm to more than 2,000 mm, whereas lowlands receive between 300 mm and 700 mm of rainfall [35]. The country’s population is estimated to exceed 120 million, with approximately 68% living in areas at risk for malaria [36].

## Registration of protocol

This systematic review was registered with the International Prospective Register of Systematic Reviews (PROSPERO). The protocol can be accessed on their registry at https://www.crd.york.ac.uk/ using the ID CRD420251127797.

## Search strategy

The searches were conducted systematically across various search engines, including Google Scholar, as well as databases such as PubMed, ScienceDirect, Scopus, and Cochrane. We focused on relevant English-language full-text articles published between July 3, 2025, and March 10, 2025. The aim was to examine the burden of malaria-HIV coinfection and associated risk factors in Ethiopia via the following key words: (“Malaria” OR “*Plasmodium*”) AND “HIV” OR “AIDS” AND (“Burden” OR “Prevalence)”) (“Co-infection” OR “coinfection” AND (“Ethiopia”). Table 2 defines the details of the search strategy for entire research databases. Studies were not restricted by publication year but were limited to English-language publications reporting on the burden and associated risk factors of malaria-HIV co-infection.

## Inclusion and exclusion criteria

Eligibility was restricted to cross-sectional studies published in English as full-text journal articles, which were independently screened by two authors (T.M. and B.A.). Essential data required for inclusion comprised confirmed co-infections, sample size, diagnostic methods, study period, and location, regardless of publication year or setting (health institution or community). Exclusion criteria were studies available only as abstracts, lacking these required attributes, and those for which the full text remained inaccessible after two email attempts to the primary authors.

## Outcome measurement

This meta-analysis aimed to estimate the overall burden of malaria-HIV co-infection and the frequency of its clinical correlates: severe anemia, hyperparasitemia, and low CD4 + T-cell counts. Outcome data were extracted into two-by-two tables using Microsoft Excel, from which the prevalence and standard error for each study were calculated. These values were then pooled using the Metan prevalence standard error command to produce the final summary estimates.

### Quality assessment and data extraction

This meta-analysis utilized the National Institutes of Health (NIH) tool to evaluate the quality of the ten included studies [37] (Table 1). All studies were included in the final synthesis, regardless of their quality scores. A structured data extraction process was implemented, in which key characteristics from each study were collected in an Excel spreadsheet under predefined categories by two authors (T.M and B.A). These characteristics included the lead author’s name, year of publication, study design, region, diagnostics, sample size, the burden of HIV-malaria co-infection, and risk factors. The reference management software EndNote (version X7.1) facilitated this process by consolidating database results and allowing for the initial identification and removal of duplicate articles.

### Data analysis

The pooled burden of malaria-HIV coinfection, along with its 95% confidence interval, was calculated via a random effects model for meta-analysis. Heterogeneity among the studies was assessed via the I² index and Cochran’s Q test, respectively. Statistical heterogeneity was assessed via Cochran’s Q test, with a p value < 0.05 considered statistically significant. I² values of 0%, 25%, 50%, and 75% represent no, low, moderate, and high heterogeneity, respectively [38]. The meta-analysis was conducted via STATA (version 16) statistical software.

## Publication bias

Publication bias was evaluated by assessing the asymmetry of the funnel plot. To distinguish true publication bias from other potential sources of asymmetry, a contour-enhanced funnel plot was created.

## Results

### Search and eligible studies

**Fig. 1:**
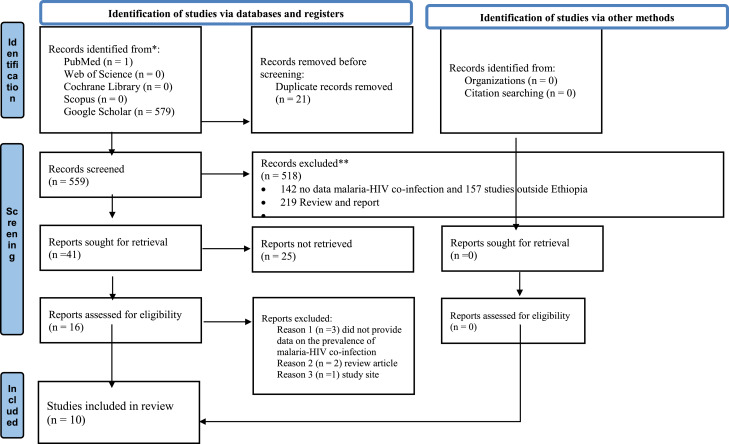
PRISMA flow diagram: Escalation of malaria-HIV coinfection and synergetic effects on malaria drug resistance in Ethiopia

From an initial pool of 580 identified records, 559 unique studies were screened by title and abstract following the removal of duplicates. Ten studies met the eligibility criteria upon full-text review and were included in the analysis. The PRISMA flow diagram (Fig. 1) illustrates the study selection process.

**Table 1:** Quality of the included studies

Reference	Selection				Compatibility	Exposure			Total score (9)
	Is the case definition adequate	Representativeness of the cases	Selection of controls	Definition of controls		Ascertainment of exposure	Same method of ascertainment for cases and controls	Non– response rate	
Kassa et al. [39]	*	*	*	*	**	*	*	*	9
Addissie et al. [28]	*	*	*	*	**	*	*	*	9
Heven [24],	*	*	*	*	**	*	*	*	9
Tegenaw et al. [40]	*	*	*	*	**	*	*	*	9
Wondimeneh et al. [41]	*	*	*	*	**	*	*	*	9
Alemayehu et al. [3]	*	*	*	*	**	*	*	*	9
Beyene et al. [25]	*	*	*	*	**	*	*	*	9
Sahle et al. [42]	*	*	*	*	**	*	*	*	9
Wondimeneh et al. [43]	*	*	*	*	*	*	*	*	8
Ejigu et al., [10]	*	*	*	*	**	*	*	*	9

*: Yes

### Features of the included studies

This systematic review and meta-analysis incorporated a total of ten articles into the final analysis [3, 10, 24, 25, 28, 39–43] (Table 2). The studies collectively involved 4,832 participants, with the smallest investigation reporting a sample size of 166 [39] and the largest study, conducted in Oromia, including 1,819 individuals [3]. Among the ten studies, nine were cross-sectional, and one was retrospective [41]. Studies were distributed across four regions: the Amhara Region contributed the most studies (*n* = 4) [25, 40, 41, 43], followed by Oromia (*n* = 3) [3, 24, 39], SNNP (*n* = 2) [10, 28], and Gambella (*n* = 1) [42]. The primary diagnostic method used in these studies was microscopy, which was employed in 80% of the studies to identify *Plasmodium* spp., aligning with its status as the gold standard for malaria diagnosis [3, 10, 28, 39–43]. Both microscopy and rapid diagnostic tests (RDTs) were employed in 2 out of 10 studies [24, 25]. For HIV identification, ELISA was used in 2 out of 10 studies [28, 39], while 5 out of 10 studies were not described (Table 2). Five studies reported severe anemia [3, 24, 25, 28, 41], three studies reported hyperparasitemia [3, 24, 42], and five studies reported a low CD4 count (≤ 200 cells/µl) [10, 24, 25, 41, 42] (see Table 3). Eight studies were reported the prevalence of *P. falciparum* [3, 24, 25, 28, 39–43], seven studies also reported about *P. vivax* and five studies were reported about mixed infection. Out of ten studies only one study was used retrospective study design [43], however nine studies were used cross-sectional study design.

**Table 2:** Characteristics of the included studies: ELISA enzyme-linked immunosorbent assay, NS not specific, RDT rapid diagnostic testing, PF. + *Plasmodium falciparum* positive, Pv. +ve *Plasmodium vivax* positive

First author	years of study	Region	Study design	Enrolled to the study	Plasmodium spp Detection	Pf. +ve	PV. +ve	Mix	HIV Detection	Positive case of MHC
Kassa et al. [39]	2002 to 2003	Oromia	Cross sectional	166	Microscope	82	81	3	ELISA and Western blot methods	8
Addissie et al. [28]	2003– 2004	SNNP	Cross-sectional	337	Microscope	337	NS	NS	ELISA	14
Heven, [24]	2007 to 2008	Oromia	Cross-sectional	500	Microscope and RDT	57	54	NS	NS	111
Tegenaw et al. [40]	2011 to 2012	Amhara	Cross-sectional	212	Microscope	6	None	None	RDT	2
Wondimeneh et al. [41]	2011 to 2012	Amhara	Retrospective	377	Microscope	53	20	None	NS	73
Alemayehu et al. [3]	2011	Oromia	Cross-sectional	1,819	Microscope	6	7	NS	KHB, STATPAK and UNIGOLD	13
Beyene et al. [25]	2012– 2013	Amhara	Cross-sectional	528	Microscope and RDT	52	37	3	NS	92
Sahle et al. [42]	2014	Gambella	Cross-sectional	172	Microscope	81	3	2	NS	86
Wondimeneh et al. [43]	2014 to 2015	Amhara	Cross- Sectional	384	Microscope	51	35	NS	KHB, STAT-PAK and Uni-gold	12
Ejigu et al. [10]	2019	SNNP	Cross-sectional	206	Microscope	NS	NS	NS	NS	103

**MHC**: malaria-HIV co-infection

**Table 3:** Severe anemia, hyperparasitaemia, and CD4 count in patients with malaria-HIV coinfection: NS: not specified

No/	Authors year	Severe anemia	Hyperparasitemia	Low CD4 count (≤ 200 cells/µL)	
1.	Kassa et al. [39]	NS	NS	NS	
2.	Addissie et al. [28]	3/14	NS	NS	
3.	Heven, [24]	20/41	37/41	26/41	
4.	Tegenaw et al. [40]	NS	NS	NS	
5.	Wondimeneh et al. [41]	4/73	NS	25/73	
6.	Alemayehu et al. [3]	8/13	2/13	NS	
7.	Beyene et al. [25]	25/92	NS	49/92	
8.	Sahle et al. [42]	NS	14/86	18/86	
9.	Wondimeneh et al. [43]	NS	NS	NS	
10.	Ejigu et al. [10]	NS	NS	5/103	

### Pooled prevalence of malaria and HIV coinfection

**Fig. 2:**
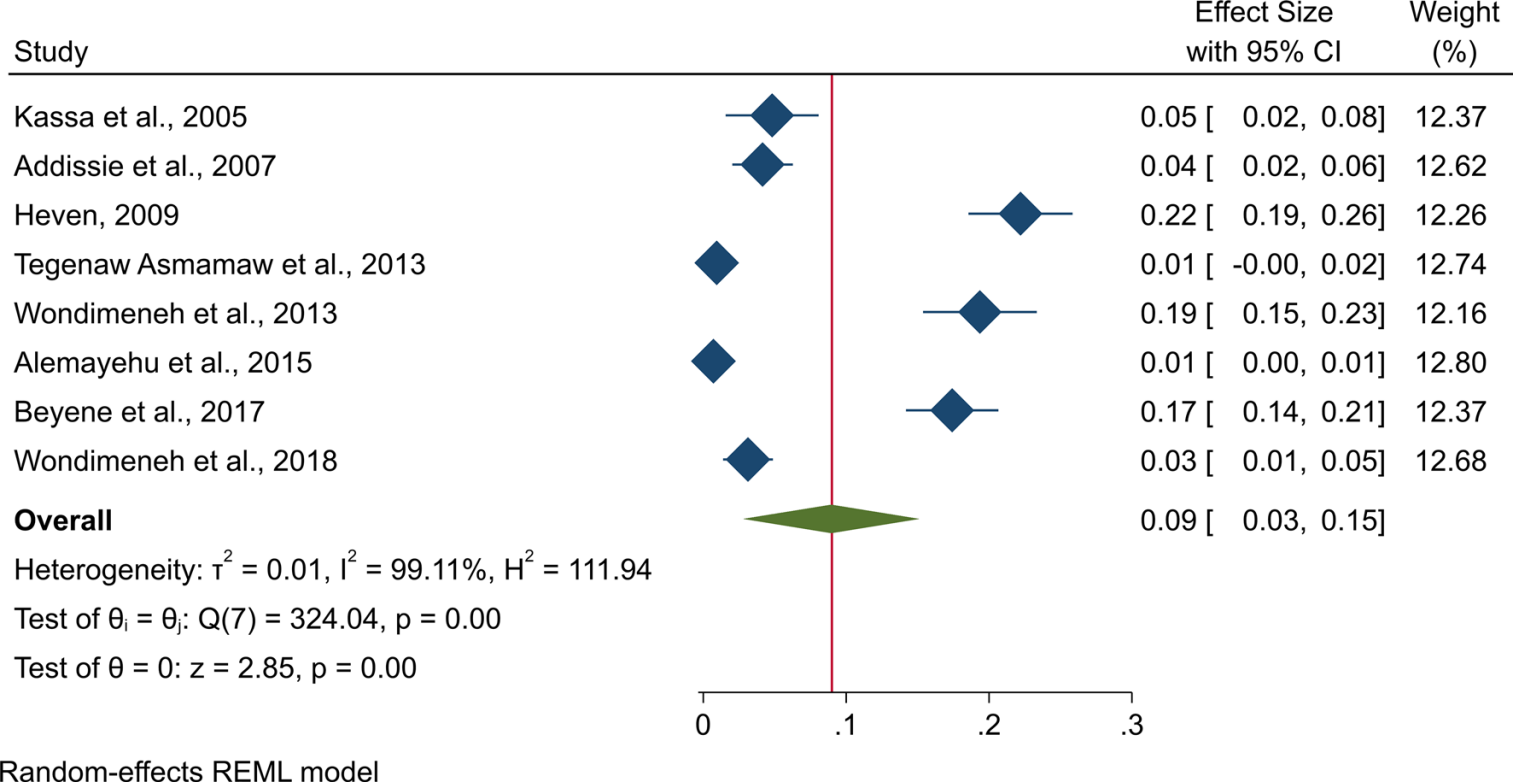
Forest plot showing the pooled prevalence estimate of malaria-HIV co-infected patients in Ethiopia: a systematic review and meta-analysis. 2025

The overall pooled prevalence of malaria co-infection with HIV, derived from eight studies using a random-effects model, was estimated at 9% (95% CI 3–15%). Significant heterogeneity was present among the study results (I^2^ =99.11%, *P* = 0.00), which is visualized in Fig. 2. The individual study estimates ranged dramatically; the highest prevalence estimate reached 50% (as published by Alemayehu et al., and Tegenaw et al. [3, 40].

### Publication bias

**Fig. 3:**
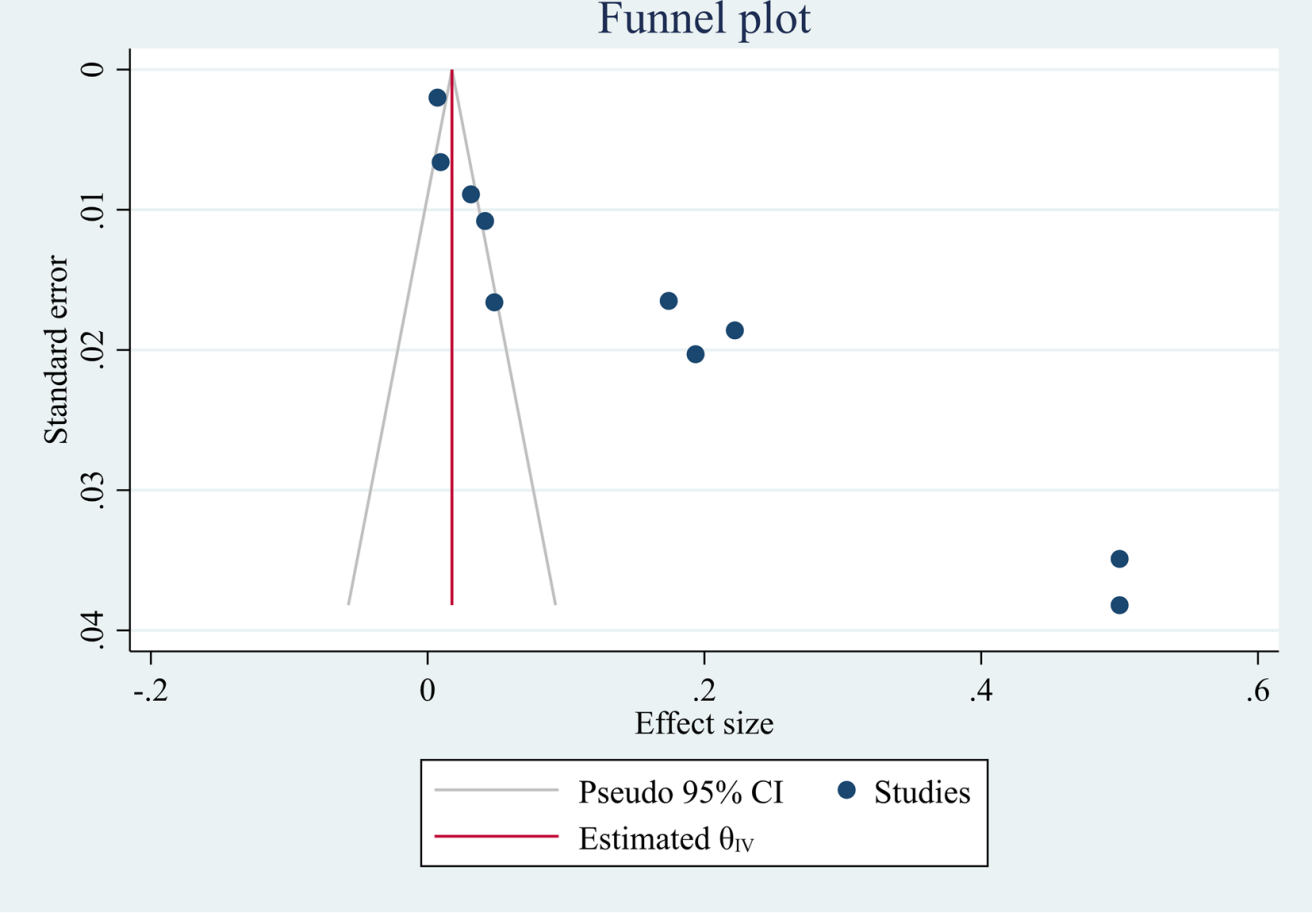
Funnel plot indicating the presence of publication bias, the increase in malaria-HIV coinfection and associated adverse outcomes in Ethiopia: A systematic review and meta-analysis. 2025

The funnel plot results (Fig. 3) initially suggested the presence of publication bias within our meta-analysis. This observation was statistically confirmed by Egger’s test, which also revealed significant evidence of bias (*P* = 0.00).

### Subgroup analysis on the basis of year of study and region of study

**Fig. 4:**
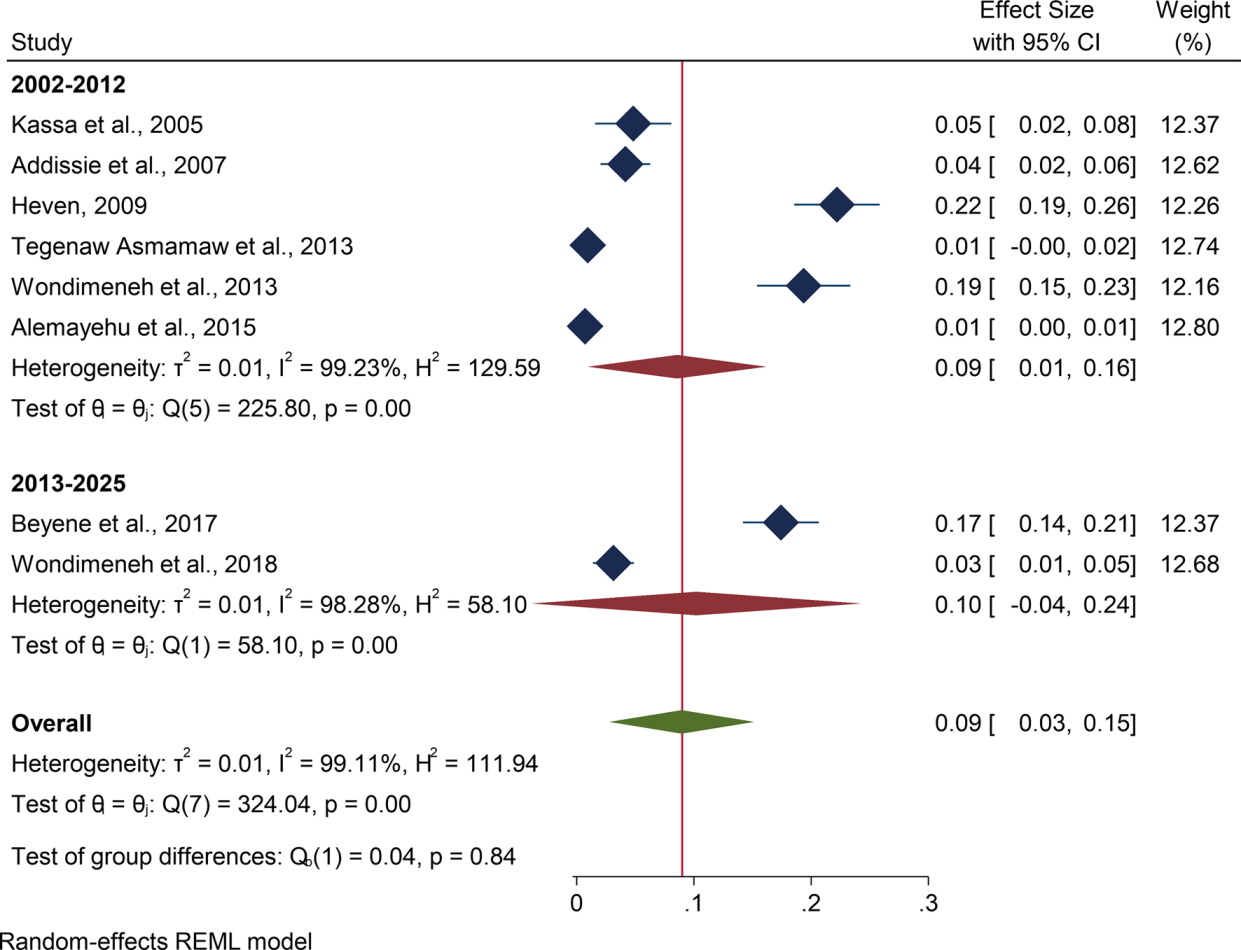
Forest plot showing the pooled prevalence estimate of malaria and HIV co-infected patients in Ethiopia: a systematic review and meta-analysis. 2025

**Fig. 5:**
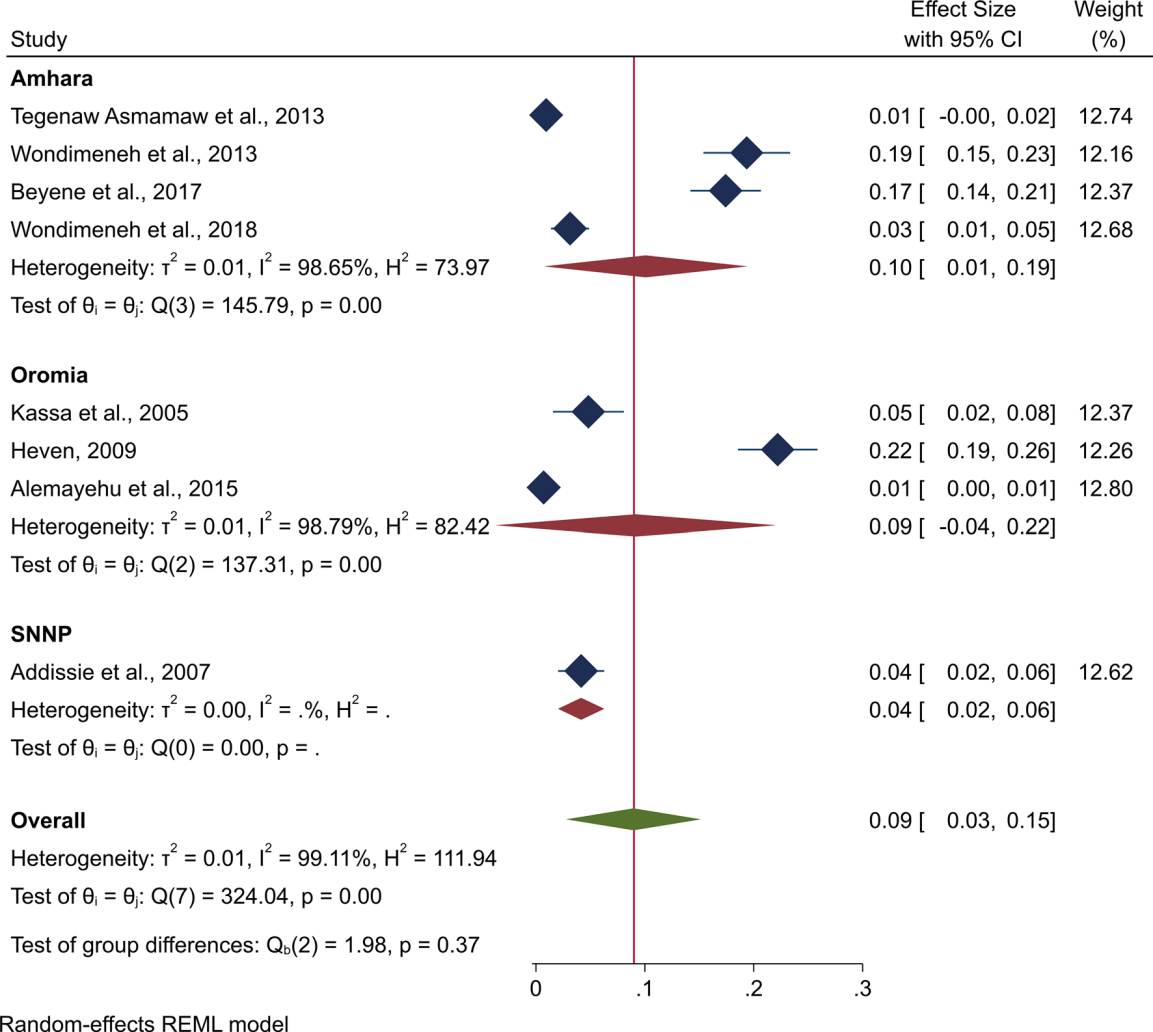
Forest plot showing the pooled prevalence estimates of malaria and HIV co-infected patients in different regions of Ethiopia: a systematic review and meta-analysis. 2025

In this meta-analysis, subgroup analysis was conducted on the basis of the year of study and the region of the study. The burden of co-infection between HIV and malaria was 9.0% (95% CI: 1.0, 16.0), according to a review of studies performed between 2002 and 2012 (Fig. 4). However, from 2013 to 2025, this prevalence increased to 10.0% (95% CI: -4, 24) (Fig. 5). The analysis in the region revealed that the highest prevalence of malaria-HIV coinfection was recorded in the Amhara Regional State at 10% (95% CI: 1.0, 19.0), followed by the Oromia Regional State at 9% (95% CI: -4.0, 22.0). The lowest prevalence was documented in the SNNP Regional State at 4% (95% CI: 2.0, 6.0) (Fig. 5).

### Severe anemia

**Fig. 6:**
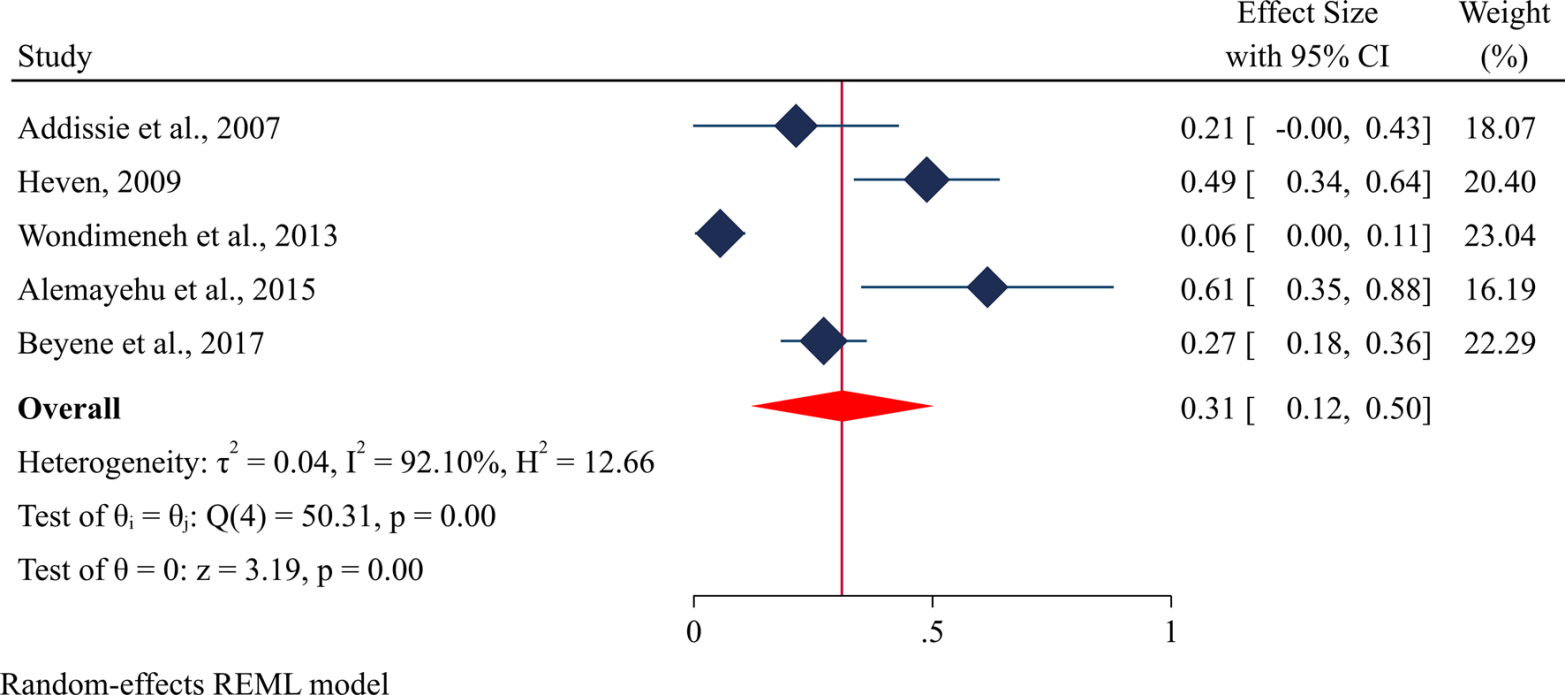
Forest plot showing the pooled prevalence estimate of severe anemia in malaria and HIV co-infected patients in Ethiopia: A systematic review and meta-analysis. 2025

The overall pooled prevalence of severe anemia in individuals with malaria and HIV coinfection was 31.0%, on the basis of data from five studies (95% CI 12.0, 50.0%) analyzed via a random effects model (Fig. 6). Among these studies, the highest prevalence estimate was 61%, reported by Alemayehu et al. [3], whereas the lowest estimate was 6%, reported by Wondimeneh et al. [41].

### Hyperparasitaemia

**Fig. 7:**
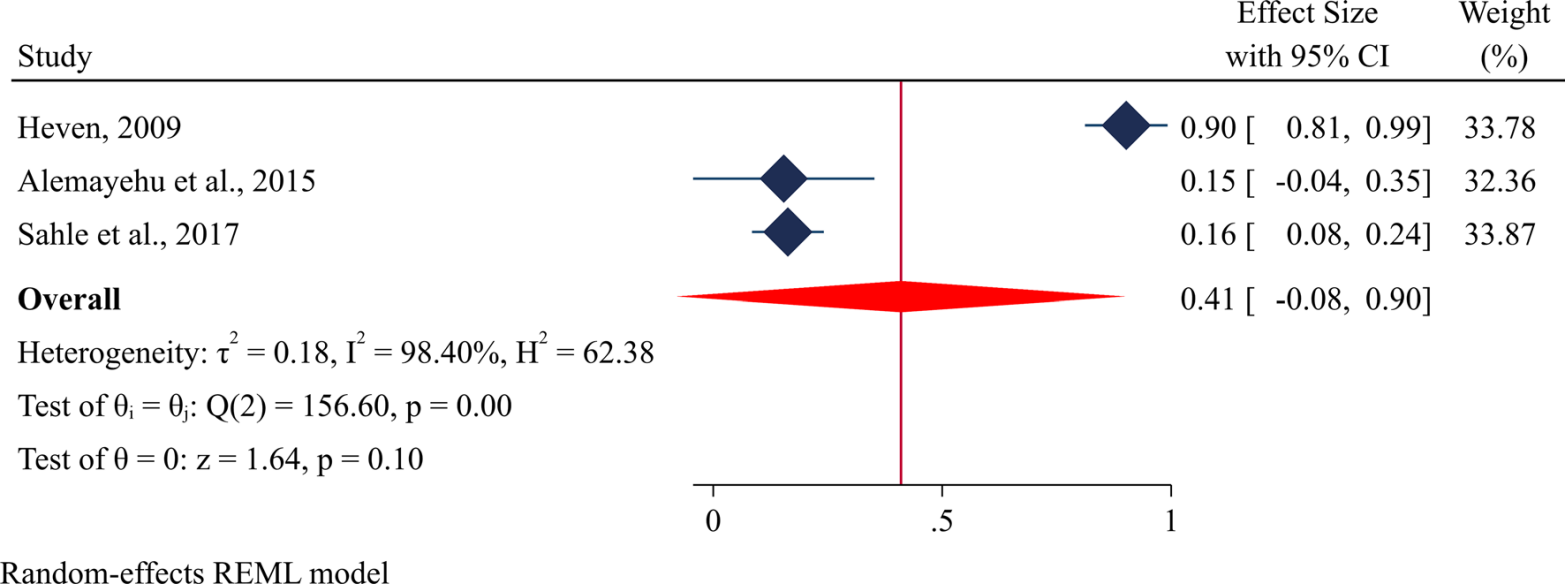
Forest plot showing the pooled prevalence estimate of hyperparasitaemia in malaria and HIV co-infected patients in Ethiopia: A systematic review and meta-analysis. 2025

According to data from three investigations, the overall pooled prevalence of hyperparasitaemia in people with HIV coinfection and malaria was 41.0% (95% CI -14.0, 0.90) (Fig. 7). Among these studies, the highest hyperparasitaemia prevalence estimate was 90%, reported by Heven et al. [24], whereas the lowest estimate was 15%, reported by Alemayehu et al. [3].

### Low CD4 count (≤ 200 cells/µL)

**Fig. 8:**
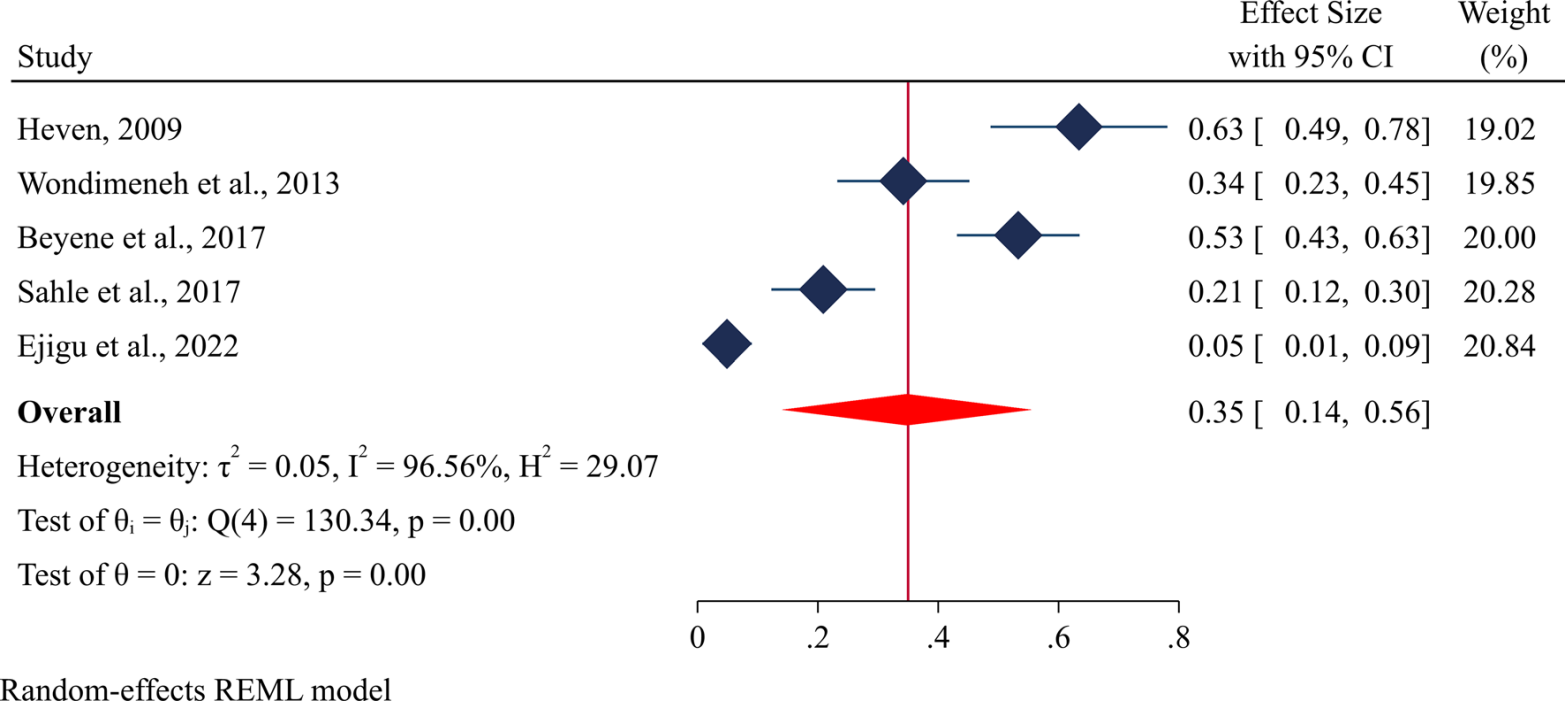
Forest plot showing the pooled prevalence estimate of low CD4 counts (≤ 200 cells/µL) in malaria and HIV-co-infected patients in Ethiopia: A systematic review and meta-analysis. 2025

The overall pooled prevalence of low CD4 counts (≤ 200 cells/µL) in individuals with malaria and HIV coinfection was 35.0% on the basis of data from five studies (95% CI 14–56%), (Fig. 8). Among these studies, Heven et al. [24], reported the highest prevalence estimate of a low CD4 count (≤ 200 cells/µL) at 63%, whereas Ejigu et al. [10], reported the lowest estimate at 5%.

## Discussion

The two deadliest illnesses, HIV and malaria, are common in Ethiopia and other parts of sub-Saharan Africa. Coinfection with HIV can impair immune responses to malaria parasites, leading to increased parasitemia [30], increased mortality rates [30, 44, 45], immunosuppression, increased susceptibility to recurrent severe episodes of malaria [19], antimalaria drug resistance [11] and reduced serum iron [46]. Similarly, in coinfection, malaria can affect both the progression of HIV [47] and the replication of viral RNA [48]. In this meta-analysis, species identification was confirmed via microscopy and RDT across nine studies. A total of 666 P. falciparum, 237 P. vivax, and 8 mixed Plasmodium infections were identified, while one study did not specify the species encountered.

The observed pooled prevalence of 9% (95% CI 3–15) in Ethiopia represents a moderate burden compared to other sub-Saharan African nations, influenced by distinct epidemiological and methodological contexts. The prevalence rates in Malawi (15%) [49], and Congo (20.33%) [32], may be attributed to similar ecological conditions that promote *Plasmodium* transmission, as well as comparable challenges in integrated HIV-malaria service delivery. Conversely, the significantly lower rates reported in Burkina Faso (3.09%) [50], and (4.4%) [51], may reflect not only lower local transmission at the time of those studies but also differences in the rigor of diagnostic tools used or the successful early implementation of universal cotrimoxazole prophylaxis, which is known to reduce malaria incidence in HIV patients. In contrast, the significantly higher prevalence found in Mozambique 72%) [52] is likely due to its intense perennial malaria transmission and a greater proportion of study participants with advanced immunosuppression (low CD4 counts), which markedly increases susceptibility to malaria. Methodologically, variations across these studies are influenced by shifting study designs ranging from community-based surveys to hospital-based cross-sectional snapshots and fluctuations in sample sizes that affect the precision of prevalence estimates. Furthermore, the specific species of *Plasmodium* predominant in a region, such as the high prevalence of *P. falciparum* in Ethiopia lowlands, interacts uniquely with the immune status of HIV-positive hosts, shaping the overall burden.

The geographical distribution of the included studies shows a clear spatial clustering of the co-infection burden (Fig. 1). Studies conducted in the regions of Amhara (10.0%) and Oromia (9.0%) reported significantly higher prevalence rates compared to SNNP (4%). As illustrated in Figure, the apparent increase in national prevalence over time aligns with a shift in research focus toward high-endemic lowland border areas in recent years.

This systematic review and meta-analysis reveal significant heterogeneity in the prevalence of malaria-HIV co-infection when studies are categorized by publication year. Between 2013 and 2025, these meta-analyses estimated a higher burden of co-infection at around 10%. In contrast, studies conducted from 2002 to 2012 generally reported lower co-infection rates or a decline in prevalence. The temporal shift in the prevalence of malaria-HIV co-infection indicates that studies conducted between 2013 and 2025 report a burden approximately 10% higher than in earlier years. This trend is likely due to improved diagnostic sensitivity, the inclusion of higher-risk populations, and a growing number of people living with HIV (PLWH) who are surviving longer due to the expanded use of antiretroviral therapy (ART), resulting in greater exposure to malaria.

The incidence of anaemia is greater in HIV-positive patients than in HIV-negative individuals because the medications used to treat HIV often contribute to elevated levels of severe anaemia [53]. Myelosuppression and malignancies associated with HIV are the main factors that trigger severe anemia [54]. Additionally, chronic inflammation caused by HIV infection and other opportunistic infections can also lead to severe anemia [55, 56]. Patients with malaria and HIV coinfection develop severe anemia, which is further worsened by a lower CD4 + T-cell count [10, 23]. Our meta-analysis revealed that the rate of severe anemia was 31 (95% CI 12–50), which is lower than the 83% and 59% reported from Mozambique [52, 57] and the 56% reported from Cameroon [53]. This is because of cotrimoxazole, which has been linked to decreased anemia in HIV-positive people, is used in conjunction with ART [58]. Our result, however, is greater than Cameroon’s 8.6% [59]. The differences may stem from various factors, including age, sex, the size of the study participants, and the rates of these two diseases. In general, coinfections can increase malaria morbidity and worsen the severity of the disease [60].

Our meta-analysis revealed that the prevalence of hyperparasitaemia was 41.0%, which is lower than the 50.7% reported in Cameron [31] and the 61% reported in Mozambique [52]. However, this finding is higher than the 7.5% reported in Nigeria [61]. The observed differences attributed to the diversity within the study populations, sample size, detection method and other associated factors.

In this systematic review and meta-analysis, the pooled prevalence of a low CD4 count (≤ 200 cells/µL) was 35%, which is consistent with the 39.2% and 28.6% reported in Cameron [31, 59] respectively. However, our result is higher than that of another study conducted in Cameroon, which reported a prevalence of only 4.9% [53].

The significant pooled prevalence and recognized risk factors underscore the necessity for comprehensive malaria-HIV screening and management protocols within clinical practice. Policy should emphasize the allocation of resources for co-endemic regions, while forthcoming research should concentrate on the cost-effectiveness of interventions and the enduring effects of coinfection.

## Conclusion

This systematic study and meta-analysis offers a comprehensive overview of the prevalence of malaria among Ethiopians living with HIV. The primary finding indicates a pooled malaria incidence of 9.0% within this population, underscoring the significant public health challenge posed by coinfection with HIV and malaria. The study emphasized two critical aspects of this coepidemic: temporal trends and notable regional disparities. Research conducted between 2012 and 2019 revealed a significantly greater prevalence than studies from 2003 to 2011 did, suggesting a rising or persistently elevated burden. This trend may reflect changes in the ecology of parasites or vectors, emerging challenges in control efforts, or the need to reassess the integration of HIV and malaria control programs.

The geographical analysis revealed that the Amhara and Oromia regions are significant hotspots for coinfection, with prevalence rates of 10% and 9%, respectively. This notable disparity compared with the lower rates observed in SNNP highlights that the burden of coinfection is not uniform across regions, necessitating targeted, region-specific interventions. Furthermore, this meta-analysis underscores the serious clinical implications of coinfection. The high pooled prevalence rates of hyperparasitemia (41%), severe anemia (31%), and advanced immunosuppression (35% with CD4 ≤ 200 cells/µL) indicate that individuals with coinfections face a substantially elevated risk of severe malaria and its associated complications. This interplay is likely to result in poorer health outcomes, increased morbidity, and higher mortality rates.

In conclusion, the co-occurrence of HIV and malaria in Ethiopia, particularly in high-burden areas, presents a complex health challenge that exacerbates both diseases. These findings highlight the urgent need for integrated service delivery that combines HIV care with effective methods for diagnosing, treating, and preventing malaria. To reduce the significant morbidity associated with this critical coinfection, public health policies must prioritize high-risk groups and geographic hotspots.

### Limitations

The pooled prevalence of malaria-HIV co-infection may be influenced by the inclusion of articles published exclusively in English. The studies included in the analysis utilized inconsistent malaria diagnostic methods, hospital-based sampling, and a small number of studies, along with inconsistent reporting of confounders such as ART status, cotrimoxazole use, pregnancy, and comorbidities. Additionally, the cross-sectional design limited the ability to determine causality or temporal sequence, which may have allowed other confounding variables to influence the outcome. Another limitation of this meta-analysis is the high heterogeneity.

## Data Availability

The data supporting the findings of this study are included in the articles.

## Abbreviations

HIV/AIDS: Human immunodeficiency virus/acquired immunodeficiency syndrome
RDT: Rapid diagnostic tests
RNA: Ribonucleic acid
SNNP: Southern nations, nationalities, and peoples’ region
WHO: World Health Organization
